# Recent advance of microbial mercury methylation in the environment

**DOI:** 10.1007/s00253-023-12967-6

**Published:** 2024-02-26

**Authors:** Xuya Peng, Yan Yang, Shu Yang, Lei Li, Liyan Song

**Affiliations:** 1 Key Laboratory of Three Gorges Reservoir Region’s Eco-Environment, Ministry of Education, Chongqing University, No. 174, Shapingba Street, Chongqing, 400045 China; 2 Division of Life Sciences and Medicine, University of Science and Technology of China, Hefei, 230026 China; 3 School of resources and environmental engineering, Anhui University, No 111 Jiulong Road, Economic and Technology Development Zone, Hefei, 230601 People’s Republic of China

**Keywords:** Mercury methylation, Functional genes, Functional strains, Driving factors, Methylation potential, Methylation mechanism

## Abstract

**Abstract:**

Methylmercury formation is mainly driven by microbial-mediated process. The mechanism of microbial mercury methylation has become a crucial research topic for understanding methylation in the environment. Pioneering studies of microbial mercury methylation are focusing on functional strain isolation, microbial community composition characterization, and mechanism elucidation in various environments. Therefore, the functional genes of microbial mercury methylation, global isolations of Hg methylation strains, and their methylation potential were systematically analyzed, and methylators in typical environments were extensively reviewed. The main drivers (key physicochemical factors and microbiota) of microbial mercury methylation were summarized and discussed. Though significant progress on the mechanism of the Hg microbial methylation has been explored in recent decade, it is still limited in several aspects, including (1) molecular biology techniques for identifying methylators; (2) characterization methods for mercury methylation potential; and (3) complex environmental properties (environmental factors, complex communities, etc.). Accordingly, strategies for studying the Hg microbial methylation mechanism were proposed. These strategies include the following: (1) the development of new molecular biology methods to characterize methylation potential; (2) treating the environment as a micro-ecosystem and studying them from a holistic perspective to clearly understand mercury methylation; (3) a more reasonable and sensitive inhibition test needs to be considered.

**Key points:**

• *Global Hg microbial methylation is phylogenetically and functionally discussed.*

• *The main drivers of microbial methylation are compared in various condition.*

• *Future study of Hg microbial methylation is proposed.*

**Graphical Abstract:**

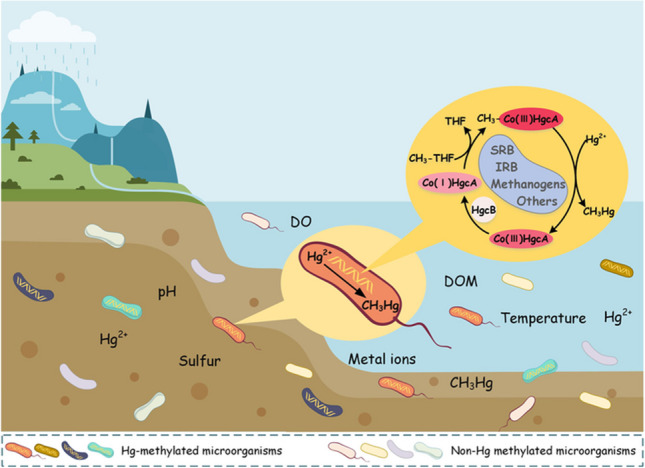

**Supplementary Information:**

The online version contains supplementary material available at 10.1007/s00253-023-12967-6.

## Introduction

Mercury (Hg), a heavy metal element, is a global pollutant that threatens the health of humans and ecosystems (Beckers and Rinklebe 2017; Driscoll et al. 2013). Methylmercury (MeHg) is one of the most biotoxic forms of Hg (Ha et al. 2017; Liu et al. 2021; Obrist et al. 2018; Stein et al. 1996). The transformation of divalent Hg (Hg^2+^) to MeHg is mainly undertaken by Hg-methylated microorganisms (Bravo et al. 2018b; Hudelson et al. 2020). Therefore, the mechanism of microbial Hg methylation has become a crucial research topic for understanding methylation in the environment and for improving the environmental risk management of Hg (Bravo and Cosio 2020; Ma et al. 2019). Numerous previous studies have shown the presence of microbial methylation of Hg in the environment, but the mechanism was unclear (Baldi 1997; Fischer et al. 1995; Ullrich et al. 2001). In 2013, the functional gene *hgcAB* of Hg methylation was reported, and the studies of Hg methylation entered the molecular level (Parks et al. 2013; Poulain and Barkay 2013).

The abundance and diversity of *hgcAB* are sensitive indicators of Hg methylation potential (Gilmour et al. 2013; Podar et al. 2015). Quantitative molecular probes of *hgcAB* genes have been developed to detect and quantify the potential of Hg-methylated microorganisms (Christensen et al. 2016). Various molecular biology techniques have also been applied to the detection and recognition of the gene *hgcAB* (Christensen et al. 2019). Molecular probes and molecular biological techniques developed were summarized and compared.

The current state of knowledge on globally isolated Hg microbial methylation strains and their associated methylation functions is reported. The potential of Hg methylation of these strains has been assessed using different indicators, which have not been uniformly standardized (Helmrich et al. 2022). Hg-methylated microorganisms reported in varied environments are also discussed here. Some strains have been reported in multiple environments, while others have been reported only in specific environments.

With regard to the biological community, we analyzed the driving factors affecting Hg methylation, including Hg^2+^ substrate, dissolved organic matter (DOM), sulfur, copper (Cu), anoxic environment, and selenium (Se) (Bravo et al. 2015; Bravo et al. 2017; Schartup et al. 2013; Tang et al. 2020). The effects of Hg bioavailability and microbial activity were analyzed and compared respectively. Microbial community interactions are also the driving factors of Hg methylation, and both intraspecific and interspecific interactions are considered and compared (Liu et al. 2019). To sum up, the driving factors and influencing mechanisms of microbial Hg methylation are urgent problems to be solved in further research.

## The functional gene of microbial Hg methylation

### Expression mechanism of gene hgcAB

**Fig. 1:**
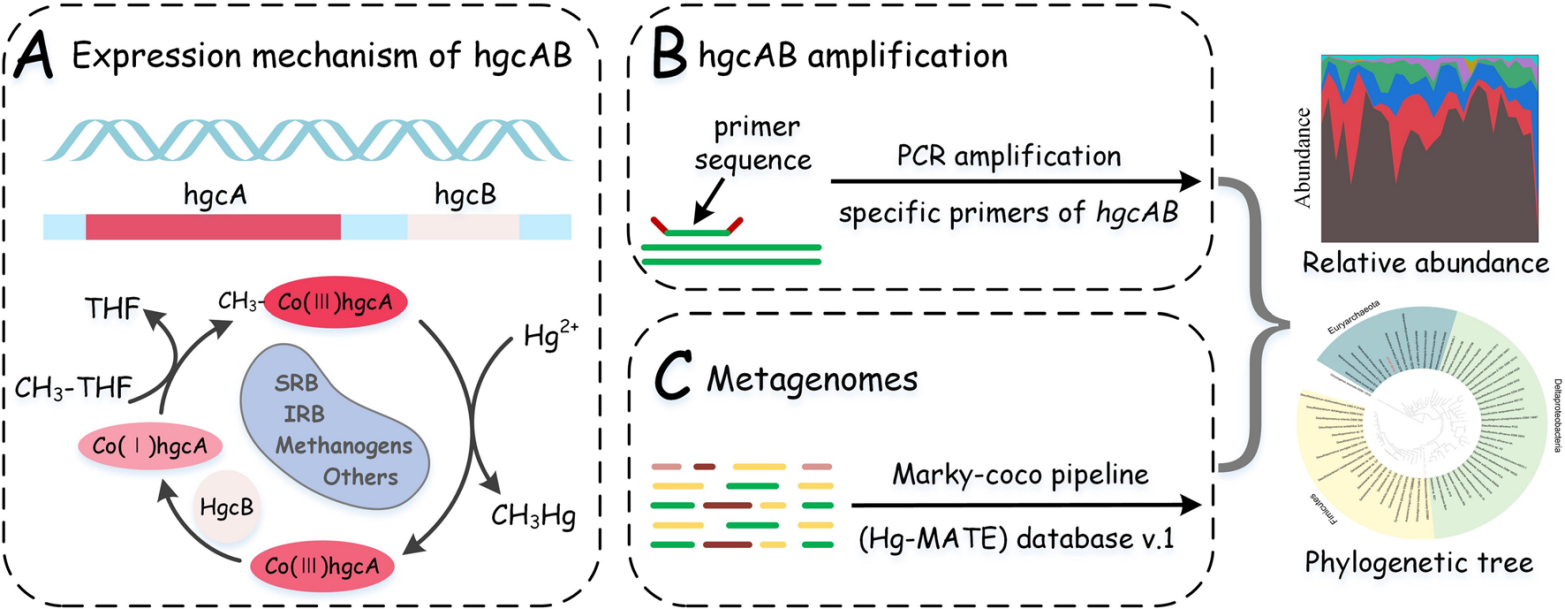
Expression mechanism (**A**) and diversity analysis (**B**, **C**) of gene *hgcAB*

The microbial Hg methylation process is carried out by methylators in the environment using the functional gene *hgcAB* to convert Hg^2+^ to MeHg (Fig. 1a). The model of the gene *hgcAB* was first proposed in 2013, in which the corrinoid protein encoded by gene *hgcA* converts Hg^2+^ to MeHg, and the corrinoid protein is reduced by the 2[4Fe-4S] ferredoxin encoded by gene *hgcB* (Parks et al. 2013). The expression mechanism of the gene *hgcAB* was further enriched in 2014, demonstrating that the corrinoid protein encoded by *hgcA* can transfer methyl groups to electrophilic substrates. Based on density functional theory, it is found that cysteine’s (Cys) thiolate coordination to corrinoid protein is conducive to the transfer of methyl radical and methyl carbanion to Hg^2+^ substrates (Zhou et al. 2013). A recent study has better described the protein structure of HgcAB through computational modeling and protein isolation. The model structure shows that there is no interaction between the two domains of HgcA; it is HgcB that forms a broad connection against the two domains, and the conserved Cys (Cys94 and Cys95) of HgcB obtain Hg^2+^ and pass it to corrinoid for methylation (Cooper et al. 2020). These studies have enriched the expression mechanism of the functional gene *hgcAB* through further verification of *hgcAB* theory and protein structure.

### HgcAB is a sensitive indicator for the potential of microbial Hg methylation

Studies have demonstrated that the presence of *hgcAB* is a reliable predictor of the Hg methylation ability of microorganisms (Gilmour et al. 2013; Podar et al. 2015). The Hg methylation rate is limited by the expression of *hgcAB* (Capo et al. 2022a). Methylation of Hg can also occur at lower protein concentrations, requiring more sensitive genetic identification methods to detect *hgcAB* in samples (Date et al. 2019).

Molecular biology methods for measuring gene abundance include metagenomic shotgun sequencing, 16S rRNA gene pyrosequencing, quantitative PCR amplification, and metaproteomics. Current common methods used to quantify *hgcAB* gene abundance include *hgcAB* PCR amplification, 16S rRNA sequencing and metagenomic sequencing. The detection depth of *hgcAB* PCR amplification is based on the development of specific primers for the *hgcAB* gene (Fig. 1b). In 2014, a variety of *hgcA* primers were designed to study *hgcA* diversity in different environments (wetland soils, paddy soils, and swamp sediments) (Bae et al. 2014; Liu et al. 2014a; Schaefer et al. 2014). The development of wide-range degenerate primers for the *hgcAB* gene was achieved in 2016, along with the development of branch-specific degenerate qPCR primers targeting three major clades (*Deltaproteobacteria*, *Firmicutes*, and *Archaea*) (Christensen et al. 2016). In addition, a new broad-range primer set for *hgcAB* and an expanded *hgcAB* reference library have been used to improve *hgcAB* amplification efficiency (Gionfriddo et al. 2020). Another way to quantify *hgcAB* is to detect, identify, and quantify *hgcAB* genes from metagenomics (Fig. 1c). According to the protocol reported by Capo et al., gene *hgcAB* in the metagenomic genome can be detected, identified, and counted through the latest *hgc* gene catalog, Hg-MATE database v1, and the marky-coco bioinformatics pipeline (Capo et al. 2022b). Comparing these methods of *hgcAB* gene detection reported above (16S rRNA sequencing, *hgcAB* PCR amplification, and metagenomic sequencing), it was found that (1) 16S rRNA gene pyrosequencing could not identify enough *hgcAB*+ species; (2) *hgcAB* clone library estimates a deeper diversity of Hg-methylators than 16S rRNA sequencing; and (3) the results from metagenomic screening showed the same diversity of *hgcAB+* microorganisms’ recognition (Christensen et al. 2019). Thus, developing new techniques that fulfill the requirements of both the diversity and specificity of *hgcAB* is urgent. Prior to the technology being updated, it is recommended that combining *hgcAB* amplification diversity and metagenomic data accurately identifies Hg-methylated microbial communities and their Hg methylation potential in the environment.

## Functional strains of Hg methylation

### Classification of Hg methylation functional strains

**Fig. 2:**
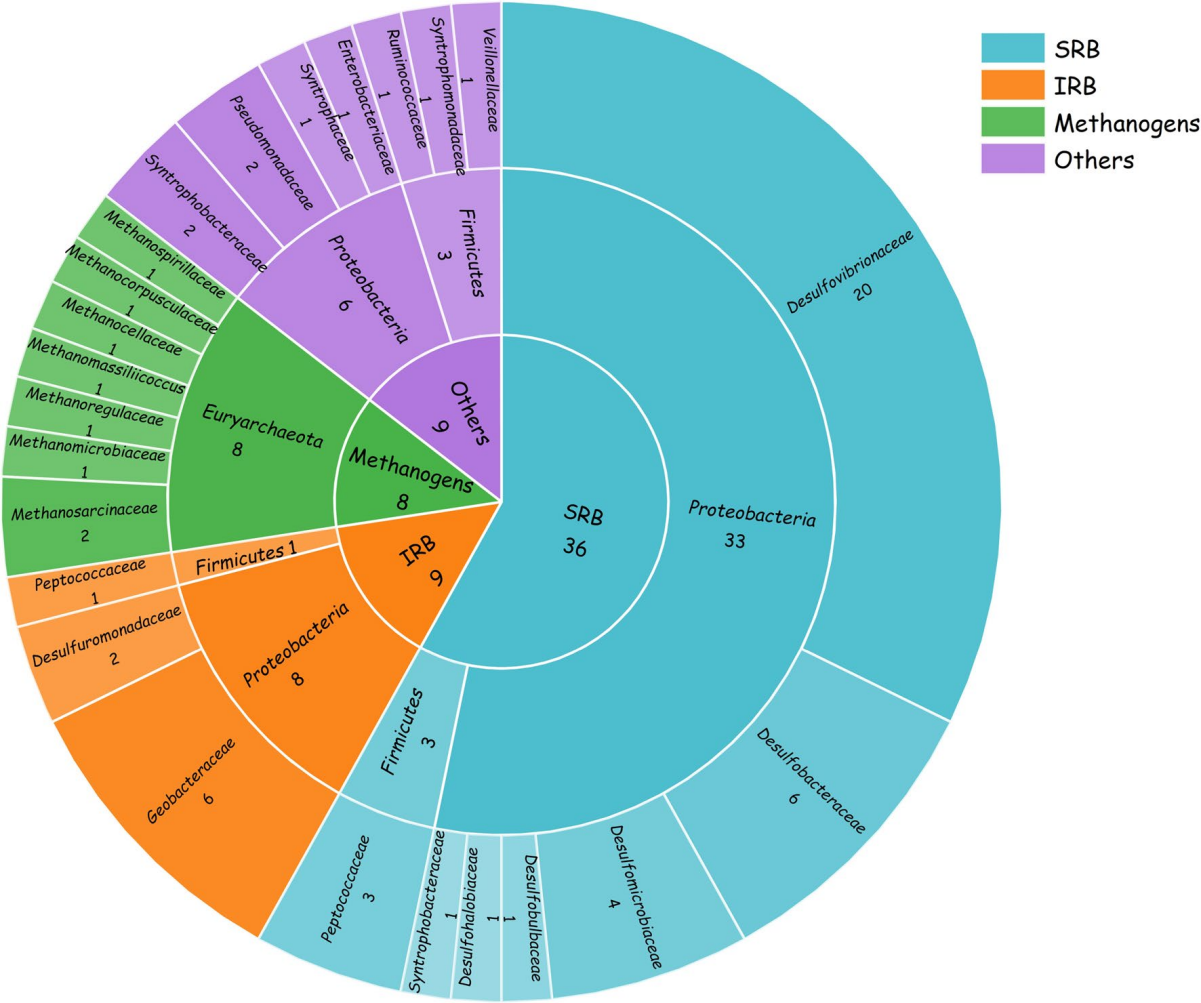
Classification and number of strains of SRBs, IRBs, methanogens, and other Hg-methylated microorganisms at phylum and family levels

The discovery of the functional gene *hgcAB* in 2013 was an essential improvement in Hg methylation mechanism research (Parks et al. 2013; Poulain and Barkay 2013). The gene cluster *hgcAB* has been used to estimate whether a strain has the potential for methylation (Christensen et al. 2016; Jones et al. 2019). In the phylogenetic distribution of genes containing the clusters *hgcAB*, methylators were reported among sulfate-reducing bacteria (SRB), iron-reducing bacteria (IRB), methanogens, and a small number of other unclassified microorganisms (Baldi 1997; Fischer et al. 1995). Although all species containing the gene *hgcAB* were distributed in these clades, not every strain in these clades has the potential for Hg methylation (Bravo et al. 2018b; Isaure et al. 2020; Liu et al. 2014b). Sixty-two strains, which had been isolated and experimentally demonstrated to have Hg methylation potential, were classified, and their methylation potential was compared (Fig. 2, Table S1). These strains included thirty-six SRBs, nine IRBs, eight methanogens, and nine other methylators. SRBs with the most significant proportion in quantity (> 60% of 62 *hgcAB+* strains) dominate microbial Hg methylation. In addition, SRB strains are primarily distributed in family *Desulfovibironaceae*, with no significant difference in the proportion of strains in the remaining families. The number of IRBs and methanogens is much smaller than that of SRBs, but the Hg methylation potential of individual strains is comparable to that of SRBs. IRB strains were mainly distributed in family *Geobacteraceae*, while the proportion of methanogens in each family was evenly distributed (Fig. 2).

Sulfate-reducing bacteria is an obligate anaerobe that uses sulfate as an electron acceptor for energy generation and uses acetic acid, lactic acid, and pyruvate as electron donors. In recent decades, SRB has been shown to contribute significantly to microbial Hg methylation in terms of quantity and Hg methylation potential. With our knowledge, 36 Hg-methylated SRB strains have been reported to date. These strains have been distributed in phylum *Proteobacteria* (33 strains) and phylum *Firmicutes* (3 strains). SRB Hg-methylators are distributed in eight families, *Desulfovibrionaceae* (20 strains), *Desulfobacteraceae* (6 strains), *Desulfomicrobiaceae* (4 strains), *Peptococcaceae* (3 strains), *Desulfobulbaceae* (1 strain), *Desulfohalobiaceae* (1 strain), and *Syntrophobacteraceae* (1 strain) (Benoit et al. 2001; Bridou et al. 2011; Brown et al. 2011; Compeau and Bartha 1985; Ekstrom et al. 2003; Feng et al. 2022; Gilmour et al. 2011; Gilmour et al. 2013; Goni-Urriza et al. 2020; Graham et al. 2012; Kerin et al. 2006; King et al. 2001; Limper et al. 2008; Lin and Jay 2007; Liu et al. 2018c; Malcolm et al. 2010; Moreau et al. 2015; Ranchou-Peyruse et al. 2009; Xiang et al. 2020; Yu et al. 2018).

Iron-reducing bacteria are members of *Deltaproteobacteria*, which use ferric iron as an electron acceptor. The role of IRBs in Hg methylation has also attracted more attention (Fleming et al. 2006). Nine IRBs have been reported to have the potential to methylate Hg, distributed in phylum *Proteobacteria* (8 strains) and phylum *Firmicutes* (1 strain). IRB Hg-methylators are distributed in three families: *Geobacteraceae* (6 strains), *Desulfuromonadaceae* (2 strains), and *Peptococcaceae* (1 strain) (Bravo et al. 2018a; Fleming et al. 2006; Graham et al. 2012; Guo et al. 2021; Warner et al. 2003).

Methanogens were the first microorganisms found to have Hg methylation potential in the early 1960s (Bravo et al. 2015; Wood et al. 1968). Nevertheless, Pak and Bartha failed to replicate the Hg methylation of methanogens in pure culture experiments in 1998, and the study of Hg methylation in methanogens has been neglected for half a century (Ma et al. 2019; Pak and Bartha 1998). Until the 2010s, inhibitory culture experiments with field samples (lake, peatlands, and sediments) suggested that methanogens might be principal methylators (Bravo et al. 2018a; Hamelin et al. 2011; Zhang et al. 2015). Subsequent studies demonstrated the Hg methylation potential of methanogens in pure culture experiments (Gilmour et al. 2018). Eight methanogens have been reported to have Hg methylation potential. They are all distributed in phylum *Euryarchaeota*. These strains are distributed in seven families, *Methanosarcinaceae* (2 strains), *Methanomicrobiaceae* (1 strain), *Methanoregulaceae* (1 strain), *Methanomassiliicoccus* (1 strain), *Methanocellaceae* (1 strain), *Methanocorpusculaceae* (1 strain), and *Methanospirillaceae* (1 strain) (Gilmour et al. 2018; Pak and Bartha 1998; Wood et al. 1968; Yu et al. 2012; Yu et al. 2013).

In addition to the microorganisms mentioned above, methylators include *fermentative*, *acetogenic*, *cellulolytic*, and other unclassified microorganisms (Gilmour et al. 2018; Gilmour et al. 2013; Jones et al. 2019). These strains were isolated from wastewaters, sediments, rice paddies, and animal guts. Nine Hg-methylated strains were reported but not attributed to SRB, IRB, or methanogens, all of which have been confirmed to carry the gene *hgcAB*. It is important to note that these strains are not all strictly anaerobic and also include facultative aerobic bacteria and aerobic bacteria (Cao et al. 2021; Feng et al. 2022). This conflicts with the current belief that Hg-methylated strains are strictly anaerobic. In the future, the role of non-anaerobic bacteria in Hg methylation is calling for more attention. Predictions of methylation potential based on the functional gene *hgcAB* expanded the number of Hg-methylators. More strains with strong Hg methylation potential are expected to be found in other species.

### Mercury methylation potential of strains

Mercury methylation potential can be characterized by the MeHg production (%MeHg), Hg methylation rate constants (*K*_m_), and normalized to protein content (picomoles of MeHg/mg protein) (Helmrich et al. 2022). In field experiments, MeHg/THg was used to represent the in situ potential for Hg methylation (Drott et al. 2008). In the laboratory studies, the variations of MeHg/THg, *K*_m_, and picomoles of MeHg/mg protein with culture time were obtained through culturing field samples to verify the methylation potential better. In addition, isotopic tracers (i.e., ^196^Hg, ^198^Hg, ^199^Hg, ^200^Hg, ^201^Hg, ^202^Hg, and ^204^Hg) were added to characterize the potential for methylation and demethylation clearly (Tang et al. 2020). Isotope labeling can allow for more accurate estimates of *K*_m_ and Hg demethylation rate constants (*K*_d_) by removing the effect of Hg compound morphology on *K*_m_. At the same time, marker elements’ localization can indicate the active transport mechanism during Hg methylation (Pedrero et al. 2012), whereas it is worth noting that the above three methods for assessing the Hg methylation potential of strains do not have a uniform standard to date.

The Hg methylation potential of Hg-methylated strains in the above classification, which has been reported in pure culture experiments and field studies, was analyzed. The Hg methylation potential of Hg-methylated strains in the above classification categories was analyzed. In SRB, strains *Desulfovibrio desulfuricans* ND132 and *Desulfovibrio caledoniensis* BerOc1 are often used as model strains to explore the mechanism of Hg methylation (Gilmour et al. 2011; Goni-Urriza et al. 2015). Studies of pure cultures showed that the methylation potential in %MeHg of strains *Desulfovibrio* sp. X2, *D. desulfuricans* ND132, and *Desulfomicrobium baculatum* X are 62.0%, 53.0%, and 34.1%, respectively (Gilmour et al. 2011; Gilmour et al. 2013) (Table S1). When picomoles of MeHg/mg protein were used to characterize Hg methylation potential, strains *D. desulfuricans* ND132 and *D. baculatum* X also have high methylation potential in picomoles of MeHg/mg protein up to 22.9 pmoles MeHg/mg protein and 26.6 pmoles MeHg/mg protein (Table S1). In IRB, strain *Geobacter sulfureducens* PCA is the model strain to study the potential of Hg methylation. The methylation potential in %MeHg of *G. sulfureducens* PCA in an iron medium reached 14.0% (Kerin et al. 2006). A laboratory study of pure cultures showed that *Geobacter daltonii* FRC-32 and *Geobacter bemidjensis* Bem were strong methylators with the methylation potential in %MeHg of 30.0% and 74.9% (Gilmour et al. 2013) (Table S1). In methanogens, *Methanospirillum hungatei* JF-1 cultured in DSM 864 has a strong potential for Hg methylation (Yu et al. 2012; Yu et al. 2013), and the methylation potential in %MeHg reached 64.2% (Gilmour et al. 2018). *Methanomassiliicoccus luminyensis* B10, *Methanosphaerula palustris* E1-9c, and *Methanocella paludicola* SANAE were confirmed to be Hg-methylators with methylation potential in %MeHg of 53.4%, 15.0%, and 8.6%, respectively (Gilmour et al. 2013; Podar et al. 2015) (Table S1). In addition, small amounts of Hg methylation strains in other classes have also shown noteworthy Hg methylation potential (Cao et al. 2021; Feng et al. 2022; Gilmour et al. 2013).

### Hg-methylated microorganisms in the environment

**Fig. 3:**
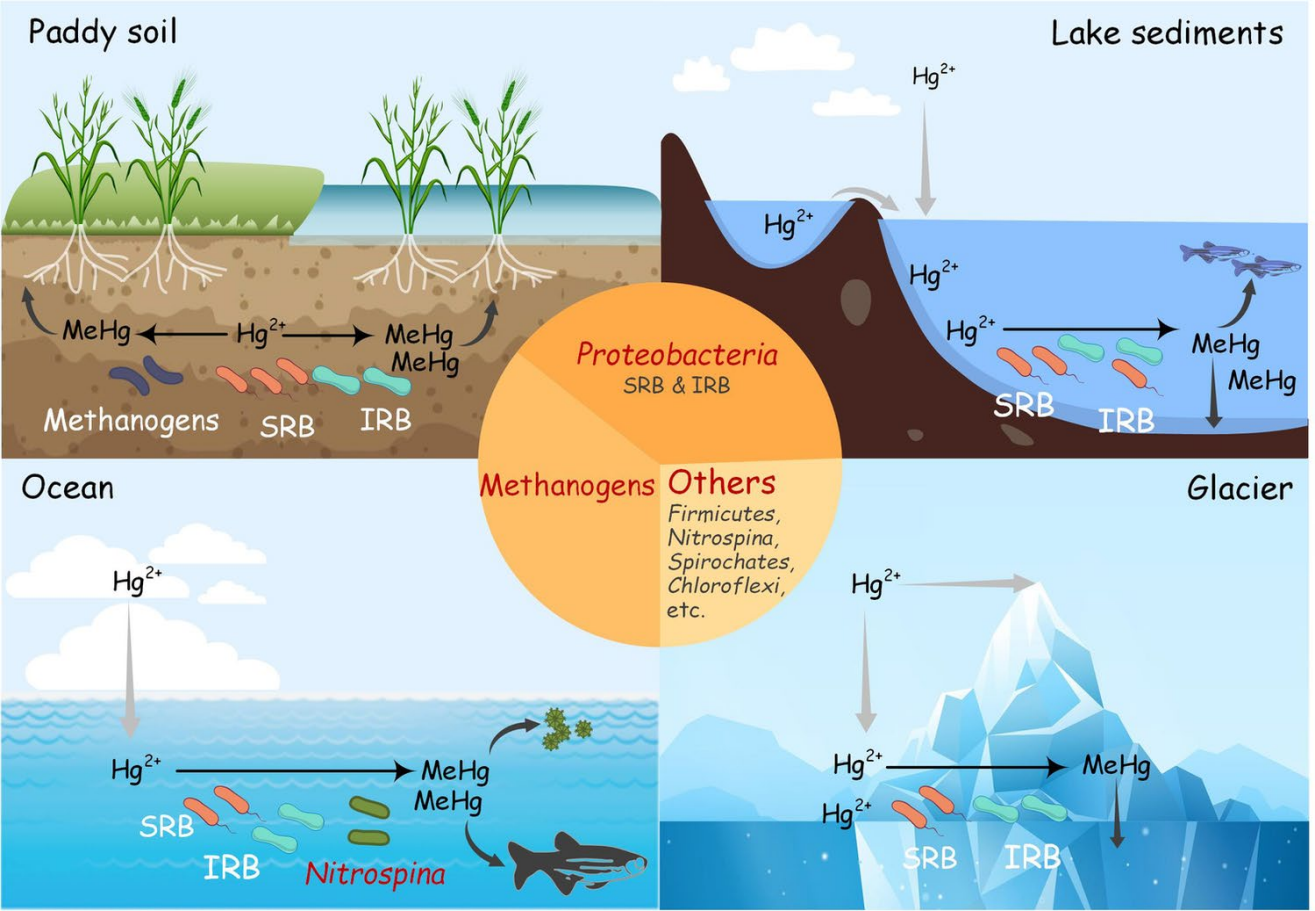
Hg-methylated microorganisms in the environments

Hg-methylated microorganisms in various environments are identified by analyzing the composition of microbial communities in field studies. *Proteobacteria* and methanogens have been widely reported in field research settings of Hg methylation. *Proteobacteria* have been reported as dominant Hg-methylated microorganisms in numerous studies on paddy soils, sediments, oceans, glaciers, and other environments (Azaroff et al. 2020; Capo et al. 2020; Lin et al. 2021; Liu et al. 2018b; Zhang et al. 2020a) (Fig. 3). In addition, when the *Proteobacteria* were further divided, SRB and IRB in proteobacteria were found to dominate microbial Hg methylation in different environmental samples. In studies on paddy soil in Guizhou, China, and sediments from 10 lakes in Spain, *Proteobacteria* SRB was found to be the largest Hg-methylated microbial community (Bravo et al. 2018a; Vishnivetskaya et al. 2018), whereas it was found that the leading Hg-methylated microorganisms belong to *Proteobacteria* IRB in two studies with sediments (Bravo et al. 2018b; Du et al. 2017). The contribution of methanogens to Hg methylation has been reported in a variety of settings, including landfills, paddy soils, and lake sediments (An et al. 2022; Jones et al. 2020; Liu et al. 2018c) (Fig. 3). In the above studies, other microbial communities (*Firmicutes*, *Chloroflexi*, *Spirochaetes*, etc.) are also involved in Hg methylation in various environments, whereas their abundance is much lower than the abundance of *Proteobacteri*a and methanogens. Specific strains have also been found in certain environments. *Nitrospina* (microaerophilic nitrite-oxidizing bacteria) is currently the only Hg-methylated microorganism reported to be present in the ocean (Tada et al. 2021; Villar et al. 2020). More environmentally representative Hg-methylated communities remain to be discovered.

## Driving factors of Hg methylation

### The bioavailability of Hg

Substrates that can be used for microbial Hg methylation include Hg^2+^, Hg complexes with natural organic matter (Hg^2+^-NOM), cinnabar (α-HgS), metacinnabar (β-HgS), Hg^2+^-complexes, and other small molecule compounds (Schaefer et al. 2011; Xiang et al. 2022; Zhang et al. 2012a). The thermodynamic stability of Hg^2+^-complexes is the main controlling factor methylation, and unstable complexes (mixed-ligation complexes containing low-molecular-mass thiol (LMM-RSH), OH–, and Cl–) have higher methylation rates than stable complexes (Hg(LMM-RS)_2_) (Adediran et al. 2019). Factors affecting the bioavailability of Hg^2+^ include DOM, sulfides, and selenium.

**Fig. 4:**
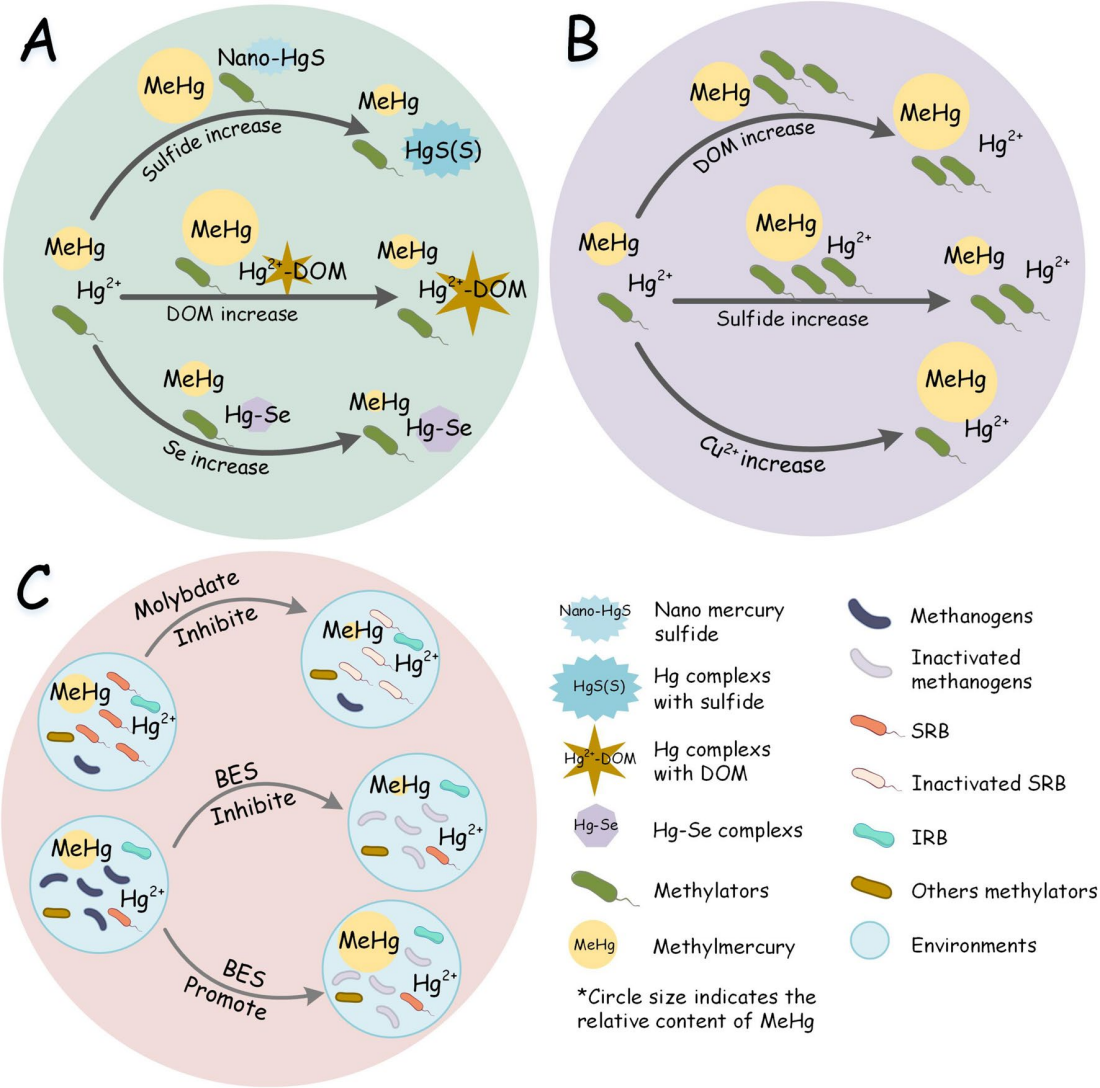
Driving factors of mercury methylation

The concentration of DOM and the combination of DOM to Hg^2+^ affect simultaneously on Hg bioavailability (Leclerc et al. 2015; Luo et al. 2017; Van et al. 2021). The concentration of DOM and the bioavailability of Hg^2+^ are inversely correlated, with low concentrations of DOM increasing the bioavailability of Hg^2+^, and high concentrations decreasing the availability of Hg^2+^ (Fig. 4a). Studies have shown that DOM with 0–0.01 mg·g^−1^ increases the bioavailability of Hg^2+^ by tenfold, compared to DOM with 0.01–0.05 mg·g^−1^ which reduces the bioavailability of Hg^2+^ (Chiasson-Gould et al. 2014). The combination of DOM and Hg^2+^ promoted the bioavailability of Hg. Most studies have focused on the binding of thiols to Hg and its affection on the bioavailability of Hg^2+^ (Bouchet et al. 2018; Thomas et al. 2020). As a special DOM molecule, thiols are the most affinity for Hg^2+^ cell ligands; thus, increasing the concentration of total thiols could promote methylation (Leclerc et al. 2015). For example, low molecular weight thiols (LMW-Thiols, such as cysteine) form Hg-thiol complexes (Hg-thiol), directly leading to the increase of MeHg production (Cardiano et al. 2011; Leclerc et al. 2015). Studies have explored the binding strength and binding law between DOM and Hg^2+^; the results show that the relative binding strength of Hg^2+^ is dimercaptopropanesulfonic (DMPS) > glutathione (GSH) > penicillamine (PEN) > cysteine > ethylenediaminetetraacetic (EDTA) > citric, acetic, and glycine, at a molar ratio of ligand-Hg < 2 (Liang et al. 2019). These results provide a new theoretical basis for studying the influence of multi-component DOM on Hg^2+^ transformation and bioavailability. In addition, DOM can slow down the precipitation of nano Hg sulfide (HgSnp), thereby improving microbial Hg methylation (Gilmour et al. 2018).

The form and size of the combination of sulfide and Hg affect its bioavailability (Fig. 4a). The increase of sulfide could inhibit microbial Hg methylation (Benoit et al. 1998; Liu et al. 2018a). Sulfides, including iron sulfide (FeS), iron disulfide (FeS_2_), carboxymethyl cellulose (CMC-FeS), and other forms of sulfides, will combine with dissolved Hg to form Hg sulfide (HgS) or Hg-complexes, and these combinations are stable (Pierce et al. 2022; Wang et al. 2020a). In addition, the morphology and binding characteristics of HgS also affect the bioavailability of Hg. HgS small molecules can be used by methylators, so the increase in HgS small molecule content has a promoting effect on Hg methylation (Li et al. 2022; Xu et al. 2021). In addition, HgS-EPS binding also increases the bioavailability of Hg, which is influenced by cell-nanoparticle interface reactions (Zhang et al. 2020b).

Recent studies have also found that antagonism between Se and Hg has an inhibitory effect on Hg methylation, mainly due to the formation of Hg-Se complexes reducing the bioavailability of Hg^2+^ (Cai et al. 2020; Truong et al. 2014; Wang et al. 2016) (Fig. 4a).

### Environmental factors affect microorganisms and microbial Hg methylation

Environmental factors drive Hg methylation by affecting the biological activity or metabolic processes of microorganisms. For substrates of microbial Hg methylation, the morphology and bioavailability of extracellular Hg^2+^ control Hg methylation by microorganisms rather than intracellular Hg^2+^ (Wang et al. 2020b). DOM promotes microbial Hg methylation because DOM provides carbon sources or nutrients for microorganisms (Bravo et al. 2017; Lei et al. 2019) (Fig. 4b). Low cysteine concentrations (0–0.06 mg·g^−1^) enhanced cell growth thus promoting methylation, whereas the growth was inhibited when the cysteine concentration reached 0.6 mg·g^−1^(Gilmour et al. 2018). The mechanism of sulfate that promotes microbial Hg methylation is that sulfate provides sufficient electron acceptors for SRB, thereby promoting SRB activity and leading to an increase in the production of MeHg (Pierce et al. 2022; Wang et al. 2018) (Fig. 4b). Moderate sulfate concentrations (0.1 mg·g^−1^) promoted the methylation significantly higher than low (0 mg·g^−1^) and high sulfate concentrations (0.5 mg·g^−1^) (Lei et al. 2021; Shao et al. 2012). In addition, copper ions (Cu^2+^) could promote methylation (Fig. 4b). The absorption of Cu^2+^ has a synergistic effect on the absorption and methylation of Hg^2+^, which is due to copper transporters or metal binding (Lu et al. 2018). Anoxic environment is more conducive to the increase and accumulation of MeHg (Yang et al. 2019). Earlier study suggested that higher MeHg production was observed in anaerobic sediments than in aerobic sediments (Olson and Cooper 1976). Since then, more studies have confirmed that microbial Hg methylation has been observed in anoxic environments (Liu et al. 2009; Mehrotra and Sedlak 2005; Warner et al. 2003). Studies indicated that the deep brine layer prevents oxygen from contacting with the DOM in the sediment and inhibits demethylation, allowing MeHg to be produced and accumulated (Valdes et al. 2017). Hg-Se antagonism could inhibit microbial Hg methylation in the environment (Dang et al. 2019). The mechanism by which selenium inhibits methylation may include (1) the formation of HgSe nanoparticle and (2) the effect of selenium on methylators or demethylators (Wright et al. 2020; Zhang et al. 2012).

### The Hg methylation potential is affected by the metabolic functions of the methylators

The classification of Hg-methylated strains includes SRB, IRB, and methanogenic bacteria (Luo et al. 2023; Ma et al. 2019). The methylation potential of microorganisms varies with their metabolic functions. There is a synergy between sulfate reduction and Hg methylation of SRB (Fig. 4c). When the sulfate reduction process was inhibited by molybdate, Hg methylation is greatly or even completely inhibited, such as 70.0% and 87.7% in peatlands and sediments, respectively (Correia and Guimaraes 2017). In addition, increasing sulfate content leads to the enhancement of SRB activity and thus promotes Hg methylation (the “Environmental factors affect microorganisms and microbial Hg methylation” section). This also demonstrated that sulfate reduction and Hg methylation are positively correlated. Many studies focus on the correlation between methanogenesis and Hg methylation of methanogens; however, there is not a clear understanding yet (Fig. 4c). Some studies suggested that the methanogenic pathway is synergistic with the Hg methylation pathway in which methane production was inhibited by 2-bromoethanesulfonate (BES) and Hg methylation is inhibited by 100% and 90% (Hamelin et al. 2011; Wang et al. 2020c), whereas a fierce competition for carbon sources and electron donors between the methanogenic and Hg methylation has also been reported, in which Hg methylation was significantly promoted in paddy soil (16.6-fold) and sediment (2-fold) when the methanogenesis was inhibited (Roth et al. 2021; Wu et al. 2020). The relationship between iron reduction and Hg methylation of IRB has been less reported. The mechanism by which iron reduction processes promote or inhibit Hg methylation remains unclear. Studies have shown that Hg methylation of IRB is not promoted when electron acceptors (FeOOH) enhance the iron reduction process (Wu et al. 2020), whereas iron reduction rates (FeRR) have a negative correlation with demethylation rates in the sediments (Avramescu et al. 2011).

### The Hg methylation potential of methylators is affected by non-methylators

Due to the complex structure of microbial communities in the environment, other strains also affect Hg methylation strains. There is a great correlation between non-Hg methylators and Hg-methylators, and studies have shown that non-Hg methylated communities play an important role in predicting Hg methylation in paddy soil (Liu et al. 2019). In addition, there are interactions between different functional strains. For example, there is a synergistic relationship between SRB and methanogens, and studies have shown that syntrophic microbial interactions dominate microbial Hg methylation (Roth et al. 2021; Yu et al. 2018).

## Perspective

### Better use of molecular biological techniques in the evaluation of methylation

The *hgcAB*+ microorganisms and community characteristics have been used to estimate the potential of Hg methylation (Bravo and Cosio 2020). Molecular biological techniques used in field studies for environmental samples include 16S rRNA, real-time PCR, high-throughput sequencing, and metagenomics (Christensen et al. 2016; Lin et al. 2023; Puglisi et al. 2019; Regnell and Watras 2019). Through statistical analyses, correlation analyses, and phylogenetic analyses of microbial community structure and *hgcAB* abundance, the dominant microbial community of Hg methylation in the environment was identified, and the mechanism of methylation by microorganisms was explored (Christensen et al. 2016). However, due to the small proportion of methylators in the total microbial community, identifying all Hg-methylated microbial communities is difficult. Therefore, molecular sequencing technology should be developed to consider both diversity and accuracy in future research. The data of microorganic diversity and metagenomic are supposed to be combined to avoid that a single index could not well represent the real situation of Hg-methylated microorganisms. In addition, the phylogenetic information for microbiota should be given more attention. First, the three-generation sequencing technology should be better used to identify Hg-methylated microorganisms (Utturkar et al. 2015). Second, newly developed high-throughput, single-microbe genomics techniques are compelling tools for characterizing the genomics information of Hg methylation strains (Zheng et al. 2022).

### Understanding methylation from microbial communities view

Field studies about the community abundance and diversity of Hg methylation microorganisms based on the functional genes *hgcAB* have been conducted. And many studies have isolated functional strains, then explored the process and mechanism of Hg methylation in the laboratory. However, to clearly understand the Hg methylation process in different environments, it is not enough to focus on the functional flora, but also to treat the environment as a micro-ecosystem and study them from a holistic perspective (Liu et al. 2019). The recommended reasons are as follows: (1) non-functional strains and functional strains may compete for limited energy sources (carbon sources, nitrogen sources, etc.) (Song et al. 2023a); (2) the metabolic of non-functional bacteria may be interrelated with the Hg methylation; (3) the abundance and proportion of functional bacteria in the ecological structure varied with environmental characteristics and non-functional bacteria. The complexity of microbial communities and interactions between communities (synergy, competition, etc.) need to be considered (Yang et al. 2021). From the perspective of microbial ecological structure, it is possible to understand the real environmental behavior mechanism of Hg more comprehensively. In addition, as with other pollutants, identifying the migration and transformation pathways of Hg and MeHg in the microbial community is also crucial for better risk management of MeHg in the environment (Song et al. 2023b; Zhang et al. 2022).

### More precise and efficient inhibitors are needed

Current research uses gene *hgcAB* as molecular markers to identify methylators in environmental samples, whereas the role of microbial communities in Hg methylation remains unclear. Inhibition culture was used to verify the contribution of microbial communities to Hg methylation (Roth et al. 2021; Wang et al. 2020c). For example, molybdate or BES was used to inhibit the sulfate-reducing pathway of SRB or the methanogenic pathway of methanogens to identify primary Hg-methylated microorganisms. However, the interpretation of inhibition culture results can be influenced by (1) complex environmental factors and (2) microbial synergies (Lei et al. 2023). Therefore, it is necessary to design a more reasonable inhibition culture experiment and consider the appropriate inhibitor gradient as well as the background concentration of substrate and electron acceptor. In addition, a more accurate interpretation of inhibitor results is needed to avoid uncertainty in evaluating the relative contributions of different microbial groups.

The complexity of the environment, the diversity and interactions of microbial communities, and the pending development of molecular biotechnology pose limitations on the study of microbial Hg methylation. Further attention needs to be paid to the simultaneous contribution of the microbial community to Hg methylation, as well as the recent application of pioneer molecular biotechnology techniques, which are important for the clear understanding of microbial Hg methylation mechanisms in the environment.

## Supplementary information

ESM 1 (PDF 227 kb)

## Data Availability

All data generated or analyzed during this study are included in this manuscript.
