# Supplementary material for: Recent advance of microbial mercury methylation in the environment
Source: Appl Microbiol Biotechnol. 2024 Feb 26;108(1):235. doi: 10.1007/s00253-023-12967-6 (PMC10896945; doi:10.1007/s00253-023-12967-6)
Supplement: Supplementary file 1 — (PDF 227 kb) [file 253_2023_12967_MOESM1_ESM.pdf]

Recent advance of microbial mercury methylation in the environment

Xuya Peng, Yan Yang, Shu Yang*, Lei Li, Liyan Song*

Supplementary Material

Content

Table S1. Confirmed mercury-methylated strains……………………………………………………………………………………Page 2-9

Table S1. Confirmed mercury-methylated strains.

metabolism	Phylum	Family	strains	*HgcAB*	number	MeHg production/ (%MeHg) [c]	pmoles MeHg/mg protein [d]	References
SRB	*Firmicutes*		*Desulfosporosinus acidiphilus* SJ4	+ [a]	DSM 22704	1.1%	1.0	(Gilmour et al., 2013)
		Peptococcaceae	*Desulfitobacterium dehalogenans*	+	DSM 9161	0.6%	0.3	(Gilmour et al., 2013)
			Desulfosporosinus youngiae	+	DSM 17734	0.6%	0.6	(Gilmour et al., 2013)
	Proteobacteria		*Desulfovibrio sp.* X2	+	N.A.[b]	62.0%	N.A.	(Gilmour et al., 2011; Gilmour et al., 2013)
		Desulfovibrionaceae	*Desulfovibrio desulfuricans* ND132	+	N.A.	53.0%	22.9	(Gilmour et al., 2011; Gilmour et al., 2013)
			Desulfovibrio alkalitolerans	+	DSM 16529	9.0%	N.A.	(Graham et al., 2012)
			Desulfovibrio aespoeensis Aspo-2	+	DSM 10631	11.0%	N.A.	(Graham et al., 2012)

	Desulfovibrio desulfuricans subsp. desulfuricans	N.A.	ATCC 3541	N.A.	N.A.	(King et al., 2001)
	Desulfovibrio desulfuricans LS	N.A.	N.A.	N.A.	N.A.	(Compeau and Bartha, 1985)
	Desulfovibrio sp. T2	N.A.	N.A.	27.0%	N.A.	(Graham et al., 2012)
	Desulfovibrio sulfodismutans ThAcO1	N.A.	DSM 3696	1.9%	N.A.	(Graham et al., 2012)
	Desulfovibrio psychrotolerans	N.A.	DSM 19430	15.0%	N.A.	(Graham et al., 2012)
	Desulfovibrio intestinalis	N.A.	DSM 11275	<0.1%	N.A.	(Limper et al., 2008)
	Desulfovibrio desulfuricans M8	N.A.	N.A.	N.A.	N.A.	(Lin and Jay, 2007)
	Desulfovibrio desulfuricans M9	N.A.	N.A.	N.A.	N.A.	(Lin and Jay, 2007)
	Desulfovibrio africanus ADR 13	N.A.	N.A.	N.A.	N.A.	(Bridou et al., 2011)

		Desulfovibrio caledoniensis BerOc1	N.A.	N.A.	N.A.	N.A.	(Bridou et al., 2011)
		Desulfovibrio sp. J2	+	KT 750867	N.A.	N.A.	(Cao et al., 2021; Gilmour et al., 2013)
		Desulfovibrio africanus str. Walvis Bay	+	ATCC 19997	N.A.	N.A.	(Brown et al., 2011)
		Desulfovibrio africanus Benghazi	+	DSM 2603	N.A.	8.0	(Ekstrom et al., 2003)
		Desulfovibrio inopinatus HHQ20	+	AF177276	1.32%	N.A.	(Goni-Urriza et al., 2020)
		Desulfovibrio longus SEBR2582	+	AY359867	0.51%	N.A.	(Goni-Urriza et al., 2020)
		Desulfovibrio oxyclinae P1B	+	U33316	0.80%	N.A.	(Goni-Urriza et al., 2020)
	Desulfobacteraceae	*Desulfosarcina variabilis* 3be13	N.A.	DSM 2060	N.A.	N.A.	(Benoit et al., 2001)
		Desulfococcus	+	DSM 2059	N.A.	N.A.	(King et al., 2001)

		multivorans 1be1					
		Desulfobacter sp. BG8	N.A.	N.A.	N.A.	N.A.	(King et al., 2001)
		Desulfobacterium sp. BG33	N.A.	N.A.	N.A.	N.A.	(King et al., 2001)
		Desulfobacterium autotrophicum HRM 2	N.A.	DSM 3382	N.A.	N.A.	(Ekstrom et al., 2003)
		Desulfobulbus propionicus MUD	N.A.	DSM 6523	N.A.	N.A.	(Ekstrom et al., 2003)
	Desulfomicrobiaceae	*Desulfomicrobium salsuginis* ADR 21	N.A.	DSM 19190	N.A.	N.A.	(Ranchou-Peyruse et al., 2009)
		Desulfomicrobium salsuginis ADR 28	N.A.	N.A.	N.A.	N.A.	(Ranchou-Peyruse et al., 2009)
		Desulfomicrobium escambiense	+	DSM 10707	N.A.	N.A.	(Feng et al., 2022)
		Desulfomicrobium baculatum X	+	DSM 4028	34.1%	26.6	(Gilmour et al., 2013)

			Desulfobulbus propionicus 1pr3	+	DSM 2032	N.A.	N.A.	(King et al., 2001; Moreau et al., 2015)
		Desulfohalobiaceae	*Desulfonatronospira thiodismutans* ASO3-1	+	DSM 19093	29.6%	N.A.	(Gilmour et al., 2013)
		Syntrophobacteraceae	*Desulfacinum hydrothermale*	N.A.	DSM 13146	N.A.	N.A.	(Malcolm et al., 2010)
IRB	*Proteobacteria*	*Geobacteraceae*	*Geobacter sulfurreducens* PCA	+	DSM 12127	14.0%	N.A.	(Kerin et al., 2006)
			Geobacter metallireducens GS-15	+	DSM 7210	5.0%	N.A.	(Kerin et al., 2006)
			Geobacter bemidjiensis Bem	+	DSM 16622	74.9%	625.0	(Gilmour et al., 2013)
			Geobacter daltonii FRC-32	+	DSM 22248	30.0%	N.A.	(Gilmour et al., 2013)
			Geobacter sp. strain CLFeRB	N.A.	N.A.	N.A.	N.A.	(Fleming et al., 2006)

		Desulfuromonadaceae	*Geobacter hydrogenophilus* H2	N.A.	DSM 13691	0.5%	N.A.	(Kerin et al., 2006)
			Geobacter anodireducens SD-1	N.A.	CP0 14963	N.A.	N.A.	(Liu et al., 2018)
			Desulfuromonas sp. DDH964	+	CPO 15080	N.A.	N.A.	(Guo et al., 2021; Liu et al., 2018)
	Firmicutes	*Peptococcaceae*	*Desulfuromonas palmitatas*	+	DSM 12391	N.A.	N.A.	(Kerin et al., 2006)
Methanogens	*Euryarchaeota*	*Methanosarcinaceae*	*Methanolobus tindarius*	+	DSM 2278	3.6%	1.5	(Gilmour et al., 2013)
			Methanomethylovorans hollandica	+	DSM 15978	3.0%	10.4	(Gilmour et al., 2013)
		Methanomicrobiaceae	*Methanofollis liminatans* GKZPZ	+	DSM 4140	0.6%	N.A.	(Gilmour et al., 2018)
		Methanoregulaceae	*Methanosphaerula palustris* E1-9c	+	DSM 19958	15.0%	N.A.	(Gilmour et al., 2018)
		Methanomassiliicoccus	*Methanomassiliicoccus luminyensis* B10	+	DSM 25720	53.4%	N.A.	(Podar et al., 2015)
		Methanocellaceae	*Methanocella paludicola* SANAE	+	DSM 17711	8.6%	N.A.	(Gilmour et al., 2018)

		Methanocorpusculaceae	*Methanocorpusculum bavaricum*	+	DSM 4179	0.2%	N.A.	(Gilmour et al., 2018)
		Methanospirillaceae	*Methanospirillum hungatei* JF-1	+	DSM 864	64.2%	0.1	(Gilmour et al., 2018; Yu et al., 2018)
Others	*Firmicutes*	*Ruminococcaceae*	*Ethanoligenens harbinense* YUAN-3	+	DSM 18485	0.9%	1.2	(Gilmour et al., 2013)
		Syntrophomonadaceae	*Dethiobacter alkaliphilus* AHT 1	+	DSM 19026	5.4%	46.4	(Gilmour et al., 2013)
		Veillonellaceae	*Acetonema longum* APO-1	+	DSM 6540	7.8%	0.9	(Gilmour et al., 2013)
	Proteobacteria	*Syntrophaceae*	*Syntrophus aciditrophicus* SB	+	DSM 26646	20.5%	191.1	(Gilmour et al., 2013)
		Pseudomonadaceae	*Pseudomonas fluorescens* TGRB2	+	KU 954349	1.4%	N.A.	(Cao et al., 2021)
			Pseudomonas putida TGRB4	-	MF 996382	0.9%	N.A.	(Cao et al., 2021; Xiang et al., 2020)
		Enterobacteriaceae	*Raoultella terrigena* TGRB3	-[e]	MK 102091	0.4%	N.A.	(Feng et al., 2022)

	Syntrophobacteraceae	*Syntrophobacter wolinii*	+	DSM 2805	N.A.	4.4	(Yu et al., 2018)
		Syntrophobacter sulfatireducens	+	DSM 16706	N.A.	N.A.	(Yu et al., 2018)

a. The *hgcAB* gene was confirmed by the molecular biological method.

b. Data not available.

c. %MeHg: The percentage of MeHg production in pure medium.

d. pmoles MeHg/mg protein: Mercury methylation potential normalized to protein content.

e. Strains do not with the *hgcAB* gene

References:

Benoit JM, Gilmour CC, Mason RP. The influence of sulfide on solid phase mercury bioavailability for methylation by pure cultures of *Desulfobulbus propionicus* (1pr3). Environmental Science & Technology 2001; 35: 127-132.

Bridou R, Monperrus M, Gonzalez PR, Guyoneaud R, Amouroux D. Simultaneous determination of mercury methylation and demethylation capacities of various sulfate-reducing bacteria using species-specific isotopic tracers. Environmental Toxicology and Chemistry 2011; 30: 337-344.

Brown SD, Wall JD, Kucken AM, Gilmour CC, Podar M, Brandt CC, et al. Genome Sequence of the Mercury-Methylating and Pleomorphic *Desulfovibrio africanus* Strain Walvis Bay. Journal of Bacteriology 2011; 193: 4037-4038.

Cao D, Chen W, Xiang Y, Mi Q, Liu H, Feng P, et al. The efficiencies of inorganic mercury bio-methylation by aerobic bacteria under different oxygen concentrations. Ecotoxicology and Environmental Safety 2021; 207: 111538.

Compeau GC, Bartha R. Sulfate-reducing bacteria - principal methylators of mercury in anoxic estuarine sediment. Applied and Environmental Microbiology 1985; 50: 498-502.

Ekstrom EB, Morel FMM, Benoit JM. Mercury methylation independent of the acetyl-coenzyme a pathway in sulfate-reducing bacteria. Applied and Environmental Microbiology 2003; 69: 5414-5422.

Feng P, Xiang Y, Cao D, Li H, Wang L, Wang M, et al. Occurrence of methylmercury in aerobic environments: Evidence of mercury bacterial methylation based on simulation experiments. Journal of Hazardous Materials 2022; 438: 129560.

Fleming EJ, Mack EE, Green PG, Nelson DC. Mercury methylation from unexpected sources: Molybdate-inhibited freshwater sediments and an iron-reducing bacterium. Applied and Environmental Microbiology 2006; 72: 457-464.

Gilmour CC, Bullock AL, McBurney A, Podar M, Elias DA. Robust Mercury Methylation across Diverse Methanogenic Archaea. Mbio 2018; 9(2): e02403-17.

Gilmour CC, Elias DA, Kucken AM, Brown SD, Palumbo AV, Schadt CW, et al. Sulfate-Reducing Bacterium *Desulfovibrio desulfuricans* ND132 as a Model for Understanding Bacterial Mercury Methylation. Applied and Environmental Microbiology 2011; 77: 3938-3951.

Gilmour CC, Podar M, Bullock AL, Graham AM, Brown SD, Somenahally AC, et al. Mercury methylation by novel microorganisms from new environments. Environ Sci Technol 2013; 47: 11810-20.

Goni-Urriza M, Klopp C, Ranchou-Peyruse M, Ranchou-Peyruse A, Monperrus M, Khalfaoui-Hassani B, et al. Genome insights of mercury methylation among *Desulfovibrio* and *Pseudodesulfovibrio* strains. Res Microbiol 2020; 171: 3-12.

Graham AM, Bullock AL, Maizel AC, Elias DA, Gilmour CC. Detailed Assessment of the Kinetics of Hg-Cell Association, Hg Methylation, and Methylmercury Degradation in Several *Desulfovibrio* Species. Applied and Environmental Microbiology 2012; 78: 7337-7346.

Guo Y, Aoyagi T, Hori T. Comparative insights into genome signatures of ferric iron oxide- and anode-stimulated *Desulfuromonas spp.* strains. Bmc Genomics 2021; 22(1): 1-17.

Kerin EJ, Gilmour CC, Roden E, Suzuki MT, Coates JD, Mason RP. Mercury methylation by dissimilatory iron-reducing bacteria. Applied and Environmental Microbiology 2006; 72: 7919-7921.

King JK, Kostka JE, Frischer ME, Saunders FM, Jahnke RA. A quantitative relationship that remonstrates mercury methylation rates in marine sediments are based on the community composition and activity of sulfate-reducing bacteria. Environmental Science & Technology 2001; 35: 2491-2496.

Limper U, Knopf B, Konig H. Production of methyl mercury in the gut of the Australian termite Mastotermes darwiniensis. Journal of Applied Entomology 2008; 132: 168-176.

Lin C-C, Jay JA. Mercury methylation by planktonic and biofilm cultures of Desulfovibrio desulfuricans. Environmental Science & Technology 2007; 41: 6691-6697.

Liu YR, Johs A, Bi L, Lu X, Hu HW, Sun D, et al. Unraveling Microbial Communities Associated with Methylmercury Production in Paddy Soils. Environ Sci Technol 2018; 52: 13110-13118.

Malcolm EG, Schaefer JK, Ekstrom EB, Tuit CB, Jayakumar A, Park H, et al. Mercury methylation in oxygen deficient zones of the oceans: No evidence for the predominance of anaerobes. Marine Chemistry 2010; 122: 11-19.

Moreau JW, Gionfriddo CM, Krabbenhoft DP, Ogorek JM, DeWild JF, Aiken GR, et al. The Effect of Natural Organic Matter on Mercury Methylation by *Desulfobulbus propionicus* 1pr3. Frontiers in Microbiology 2015; 6.

Podar M, Gilmour CC, Brandt CC, Soren A, Brown SD, Crable BR, et al. Global prevalence and distribution of genes and microorganisms involved in mercury methylation. Science Advances 2015; 6: 1389.

Ranchou-Peyruse M, Monperrus M, Bridou R, Duran R, Amouroux D, Salvado JC, et al. Overview of Mercury Methylation Capacities among Anaerobic Bacteria Including Representatives of the Sulphate-Reducers: Implications for Environmental Studies. Geomicrobiology Journal 2009; 26: 1-8.

Xiang Y, Wang Y, Shen H, Wang D. The Draft Genome Sequence of *Pseudomonas putida* Strain TGRB4, an Aerobic Bacterium Capable of Producing Methylmercury. Curr Microbiol 2020; 77: 522-527.

Yu RQ, Reinfelder JR, Hines ME, Barkay T. Syntrophic pathways for microbial mercury methylation. ISME J 2018; 12: 1826-1835.
